# Indwelling versus Intermittent Urinary Catheterization following Total Joint Arthroplasty: A Systematic Review and Meta-Analysis

**DOI:** 10.1371/journal.pone.0130636

**Published:** 2015-07-06

**Authors:** Wei Zhang, An Liu, Dongcai Hu, Deting Xue, Chao Li, Kai Zhang, Honghai Ma, Shigui Yan, Zhijun Pan

**Affiliations:** 1 Department of Orthopedics, Second Affiliated Hospital, School of Medicine, Zhejiang University, Hangzhou, People’s Republic of China; 2 Department of Surgery, Second Affiliated Hospital of Zhejiang University School of Medicine, Hangzhou, Zhejiang Province, People’s Republic of China; 3 Department of Nuclear Medicine, Second Affiliated Hospital of Zhejiang University School of Medicine, Hangzhou, Zhejiang Province, People’s Republic of China; 4 Department of Thoracic surgery, First Affiliated Hospital of Zhejiang University School of Medicine, Hangzhou, Zhejiang Province, People’s Republic of China; Institute of Biochemistry and Biotechnology, TAIWAN

## Abstract

**Objective:**

The purpose of this study is to compare the rates of urinary tract infection (UTI) and postoperative urinary retention (POUR) in patients undergoing lower limb arthroplasty after either indwelling urinary catheterization or intermittent urinary catheterization.

**Methods:**

We conducted a meta-analysis of relevant randomized controlled trials (RCT) to compare the rates of UTI and POUR in patients undergoing total joint arthroplasty after either indwelling urinary catheterization or intermittent urinary catheterization. A comprehensive search was carried out to identify RCTs. Study-specific risk ratios (RR) with 95% confidence intervals (CI) were pooled. Additionally, a meta-regression analysis, as well as a sensitivity analysis, was performed to evaluate the heterogeneity.

**Results:**

Nine RCTs with 1771 patients were included in this meta-analysis. The results showed that there was no significant difference in the rate of UTIs between indwelling catheterization and intermittent catheterization groups (*P*>0.05). Moreover, indwelling catheterization reduced the risk of POUR, versus intermittent catheterization, in total joint surgery (*P*<0.01).

**Conclusions:**

Based on the results of the meta-analysis, indwelling urinary catheterization, removed 24-48 h postoperatively, was superior to intermittent catheterization in preventing POUR. Furthermore, indwelling urinary catheterization with removal 24 to 48 hours postoperatively did not increase the risk of UTI. In patients with multiple risk factors for POUR undergoing total joint arthroplasty of lower limb, the preferred option should be indwelling urinary catheterization removed 24-48 h postoperatively.

**Level of Evidence:**

Level I.

## Introduction

Because of the aging of population and the improvements in medical technology, there has been a major increase in the number of joint arthroplasty over the past two decades. According to a recent survey, the rate of joint arthroplasty increased by 59.4% from 1991 to 2010, that is, from 3.2 to 5.1 per 10,000 people [1]. However, the rapid growth in the number of surgeries performed has also coincided with a considerable increase in postoperative complications. Postoperative urinary retention (POUR) is a common complication following lower joint arthroplasty, and it occurs in 0–75% of patients [2]. The overall rate of POUR in the general surgical population is about 3.8%, while the rate after lower limb arthroplasty is as much as 20-folder higher [3]. Moreover, POUR has been associated with the development of urinary tract infection (UTI), and the subsequent risk of wound and implant infection [4]. It has been considered to be a predisposing factor for the development of subsequent periprosthetic joint infection [5]. Additionally, the sequelae of POUR can delay early mobilization, prolong hospitalization, and increase readmission rates [6]. Thus, for orthopaedic surgeons and nurses, the preventing POUR in total joint arthroplasty constitutes a Gordian knot of post-operative complications [7].

Although various approaches to prevent POUR have been taken, the results were inconclusive. To date, therefore, indwelling and intermittent urinary catheterization are the only options for the prevention and treatment of POUR [7]. Nevertheless, there remains a conflict regarding these two bladder management protocols. Some researches recommended that indwelling catheterization might be a valid and reasonable option, since it was more effective in preventing POUR [8–11] and did not increase the incidence of UTI [8–11], compared with intermittent catheterization. Moreover, indwelling urinary catheterization has often been used in longer operative procedures in order to monitor urinary output and guide fluid resuscitation, which is conducive to ensuring patient status. In contrast, others have indicated that preoperative indwelling catheterization is an unnecessary routine practice for patients undergoing total joint arthroplasty. With improvements in surgical and anesthetic technologies, the incidence of POUR is relatively low even without the use of an indwelling catheter in patient undergoing total joint arthroplasty [10]. Intermittent urinary catheterization could be similarly effective in preventing POUR. Furthermore, the use of intermittent catheterization reduced the risk of UTI [12–14]. So unnecessary urinary catheterizations could be avoided altogether in many patients, contributing to earlier rehabilitation.

Several randomized controlled trials (RCT) of optimal bladder management have been published [8–10, 12, 14–18], but whether routine indwelling catheterizations should be performed remains controversial. Therefore, a quantitative meta-analysis is required to evaluate and summarize the issue and provide evidence for clinical setting. The purpose of this study is to compare the rates of UTI and POUR in patients undergoing lower limb arthroplasty after indwelling or intermittent urinary catheterization.

## Materials and Methods

This meta-analysis was performed according to the guidelines for ‘preferred reporting items for systematic reviews and meta-analyses’ (the ‘PRISMA’ statement) [19].

### Data retrieval strategies

Electronic databases, including PubMed, Embase, and the Cochrane Library, were searched by two independent researchers (WZ and AL). Data were last updated on 5 January 2015. The following keywords or corresponding Medical Subject Headings (MeSH) were used: “catheter” or “catheterization” or “catheterize” and “total knee arthroplasty” or “total knee replacement” or “total hip replacement” or “total hip arthroplasty” or “TKA” or “TKR” or “THR” or “THA” or “total joint replacement” or “total joint arthroplasty” or “TJA” or “TJR”. Details of the search strategy are provided in S1 Table and S1 Text. Reference lists of the relevant articles were also reviewed for any additional relevant studies. The search was not restricted by language.

### Inclusion criteria

Studies were identified according to the following inclusion criteria: 1) Participants: human with relevant diseases requiring surgical interventions, 2) Intervention: primary total joint arthroplasty in the lower limbs, 3) Comparison: patients with intermittent catheterization versus those with indwelling catheterization. Patients in the indwelling urinary catheterization group received an indwelling catheter before surgery and the catheter was removed within 48 h postoperatively. Patients in the intermittent urinary catheterization group did not receive a urinary catheter before surgery, and a one-time catheterization was provided if the patient had the urge to void but was unable to urinate, 4) Outcome: trials that reported important postoperative outcomes, such as UTI and POUR (at least one outcome), and 5) Methodological criterion: a prospective RCT.

The following exclusion criteria were used: 1) insufficient data were available to estimate a risk ratio (RR), 2) animal studies and cadaver studies, and 3) the size of each group in the RCT was less than 10.

### Data extraction

Two authors (WZ and AL) extracted relevant data independently, including the first author’s name, study region, study design, publication year, the size of indwelling and intermittent urinary catheterization groups, average age of participants, gender ratio, type of anesthesia, duration of indwelling catheterization, antibiotic prophylaxis, surgical site (knee or hip), UTI, and POUR. UTI was defined as a urine sediment positive for bacteria or white blood cells with a positive urine culture of >100,000 colonies. Intention-to-treat (ITT) data gathered from the studies were used as long as it was available. Otherwise, we used data from the analysis of the available data or data from the analysis of treatment received.

### Quality assessment

According to the 12-item scale [20], the methodological quality of each included RCT was assessed by two independent researchers (WZ and AL). The 12-item scale consisted of the followings: randomized adequately, allocation concealed, patient blinded, care provider blinded, outcome assessor blinded, acceptable dropout rate, ITT analysis, avoided selective reporting, similar baseline, similar or avoided cofactor, patient compliance and similar timing. Disagreements were evaluated using a kappa test and consensus was achieved by discussion with the corresponding author (ZJP).

### Statistical analysis

Statistical analyses were performed using the Stata software (ver. 12.0; StataCorp LP, College Station, TX, USA). The relative risk (RR), with corresponding 95% confidence intervals (CI), was considered to be the effect estimate for all included studies. Depending on the level of heterogeneity, study-specific RRs were pooled using a fixed-effect model or a random-effects model. Statistical heterogeneity was assessed with the Q-test and *I* ^*2*^. *I* ^*2*^ values of 25%, 50%, and 75% were considered to indicate low, moderate, and high heterogeneity, respectively [21]. If *P*>0.1 and *I* ^*2*^<50%, a fixed-effect model was used; otherwise, a random-effect model was used. For moderate and high heterogeneity, a meta-regression analysis was conducted, based on methodological quality or clinical diversity (e.g., study region, publication year) to identify the origin of the heterogeneity among the studies. A sensitivity analysis (backward elimination stepwise regression analysis) was also conducted by omitting one study at a time and examining the influence of each study individually. Egger’s test and Begg’s test were performed to assess the publication bias. For all statistical analyses, with the exception of heterogeneity, a value of *P*<0.05 was considered to indicate statistical significance, and all tests were two-sided.

## Results

### Study selection

**Fig 1:**
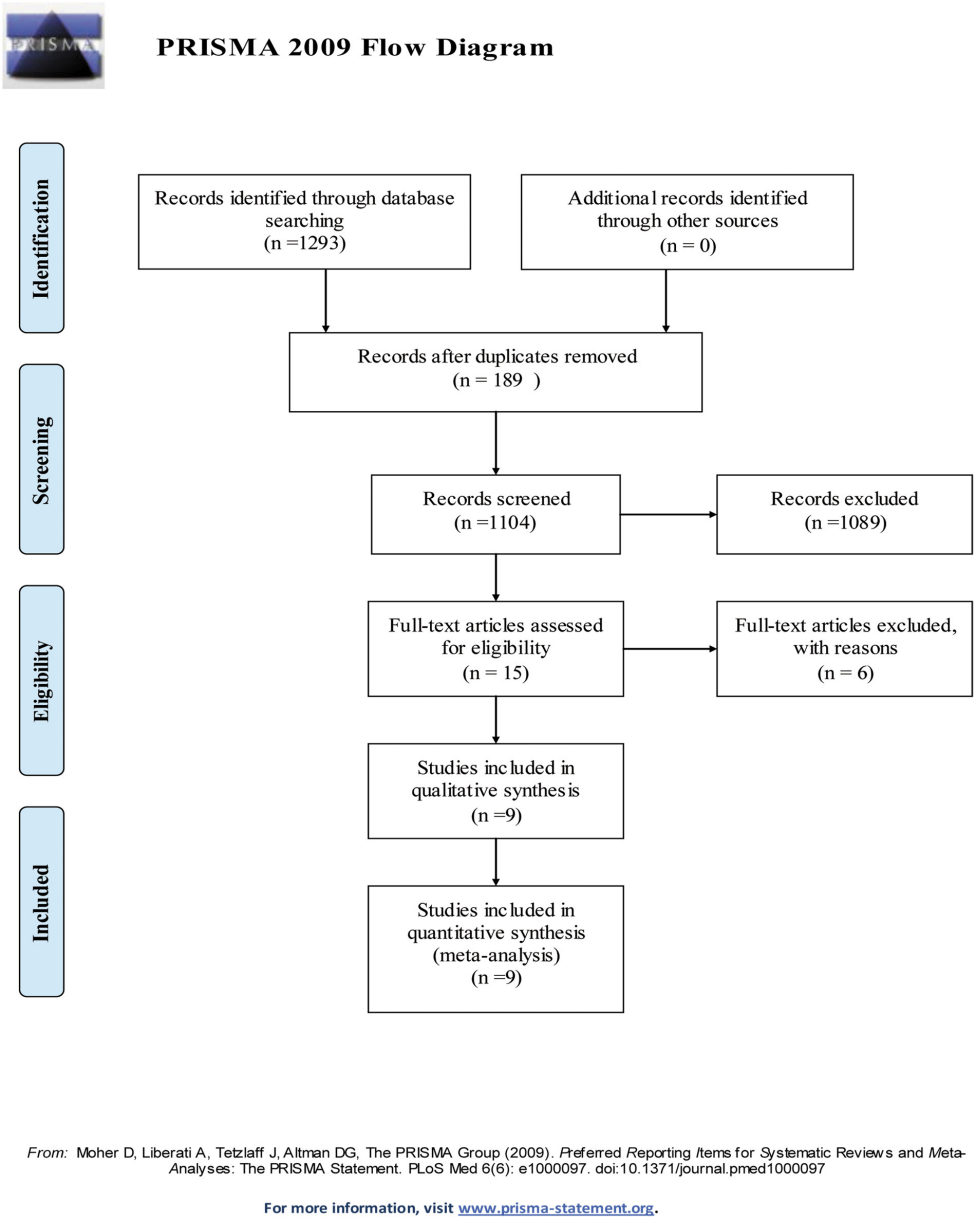
Flow chart summarizing the selection process of studies.

The process for selecting studies is shown in Fig 1. The search yielded 1293 potentially relevant articles: 293 from PubMed, 832 from Embase, and 168 from the Cochrane library. Of these, 189 studies were excluded as duplicates. After viewing the titles and abstracts of the 1104 remaining studies, the full texts of 15 studies were retrieved. Two studies were not RCTs [22, 23]. Because sufficient data were not available in three studies, they were excluded [11, 24, 25]. One ‘study’ was also excluded because it was being an editorial [7]. Finally, nine RCTs were included in this study [8–10, 12, 14–18].

### Study characteristics

The characteristics of the nine included studies are presented in Table 1: five were from the United States, three were from European countries, and one was from an Asian country. The dataset consisted of 1771 patients: 870 in the indwelling catheterization group and 901 in the intermittent catheterization group. A dropout rate of ≦10% was accepted in each trial. The average age, gender ratio and surgical site were also noted. All indwelling catheters were removed within 24–48 h postoperatively. The demographic baselines of the two groups in each included RCT were comparable.

**Table 1:** Studies used for the meta-analysis.

First author	Publication year	Region	Group size		Dropout rate	Average age (yr)		Gender ratio (male/female)		Surgical site (knee/hip)		Anesthesia	Catheterization time^1^	Antibiotic prophylaxis
			Indwelling	Intermittent		Indwelling	Intermittent	Indwelling	Intermittent	Indwelling	Intermittent			
Brand [12]	2001	Netherlands	46	53	0**%**	68.6	68.2	13/33	14/39	26/20	35/38	General/Spinal	24 h	Y
Halleberg [15]	2013	Sweden	89	93	10**%**	71.9	72.1	36/49	37/48	0/89	0/93	General/Spinal	48 h	Unclear
Huang [14]	2014	China	157	157	0**%**	66.9	67.4	33/124	38/119	157/0	157/0	General	24 h	Unclear
Knight [8]	1996	USA	62	57	0**%**	66.0	66.0	28/34	29/28	22/40	20/37	Unclear	48 h	Y
Michelson [17]	1988	USA	41	55	0**%**	65.7	61.7	Unclear	Unclear	21/23	22/34	Unclear	24 h	Unclear
Miller [10]	2013	USA	107	93	0**%**	60.1	58.7	50/57	52/51	0/107	0/93	Spinal	48 h	Unclear
Lampe [9]	1992	**N**etherlands	39	24	0**%**	Unclear	Unclear	Unclear	Unclear	0/39	0/24	Unclear	48 h	Unclear
Iorio [16]	2000	USA	306	346	0**%**	67.8	66.8	142/163	162/184	306/0	346/0	General/Spinal	24 h	Unclear
Carpiniello[18]	1988	USA	23	23	0**%**	70	70	0/23	0/23	13/10	9/14	Unclear	24 h	Unclear

^1^ the duration of indwelling catheterization

### Study quality

Table 2 shows the quality of the included studies. The average score of the quality assessment of the included studies was 8.33. Of these, four studies were high quality, which explicitly introduced randomized method, concealed allocation, and the blinding method. The other five studies achieved moderate quality. There was an excellent interrater agreement between investigators on the eligibility (*Κ* = 0.78).

**Table 2:** Study quality.

First author	Randomized adequately^1^	Allocation concealed	Patient blinded	Care provider blinded	Outcome assessor blinded	Acceptable drop-out rate^2^	ITT Analysis^3^	Avoided selective reporting	Similar baseline	Similar or avoided cofactor	Patient compliance	Similar timing	Quality^4^
Brand [12]	No	No	No	No	No	Yes	Yes	Yes	Yes	Yes	Yes	Yes	Moderate
Halleberg [15]	Yes	Yes	No	No	Yes	Yes	Yes	Yes	Yes	Yes	Yes	Yes	High
Huang [14]	Yes	Yes	Yes	Yes	No	Yes	Yes	Yes	Yes	Yes	Yes	Yes	High
Knight [8]	No	No	No	No	No	Yes	Yes	Yes	Yes	Yes	Yes	Yes	Moderate
Michelson [17]	Yes	No	No	No	Yes	Yes	Yes	Yes	Yes	Yes	Yes	Yes	High
Miller [10]	Yes	No	Yes	Yes	No	Yes	Yes	Yes	Yes	Yes	Yes	Yes	High
Lampe [9]	No	No	No	No	No	Yes	Yes	Yes	Yes	Yes	Yes	Yes	Moderate
Iorio [16]	No	No	No	No	No	Yes	Yes	Yes	Yes	Yes	Yes	Yes	Moderate
Carpiniello [18]	No	No	No	No	No	Yes	Yes	Yes	Yes	Yes	Yes	Yes	Moderate

^1^ Only if the method of sequence made was explicitly introduced could get a ‘Yes’.

^2^ Drop-out rate <20% could get a ‘Yes’, otherwise ‘No’.

^3^ ITT = intention-to-treat, only if all randomized participants were analyzed in the group they were allocated to could receive a ‘Yes’.

^4^ “Yes” items more than 7 means ‘High’; more than 4 but no more than 7 means ‘Moderate’; no more than 4 means ‘Low’.

### Meta-analysis results

**Fig 2:**
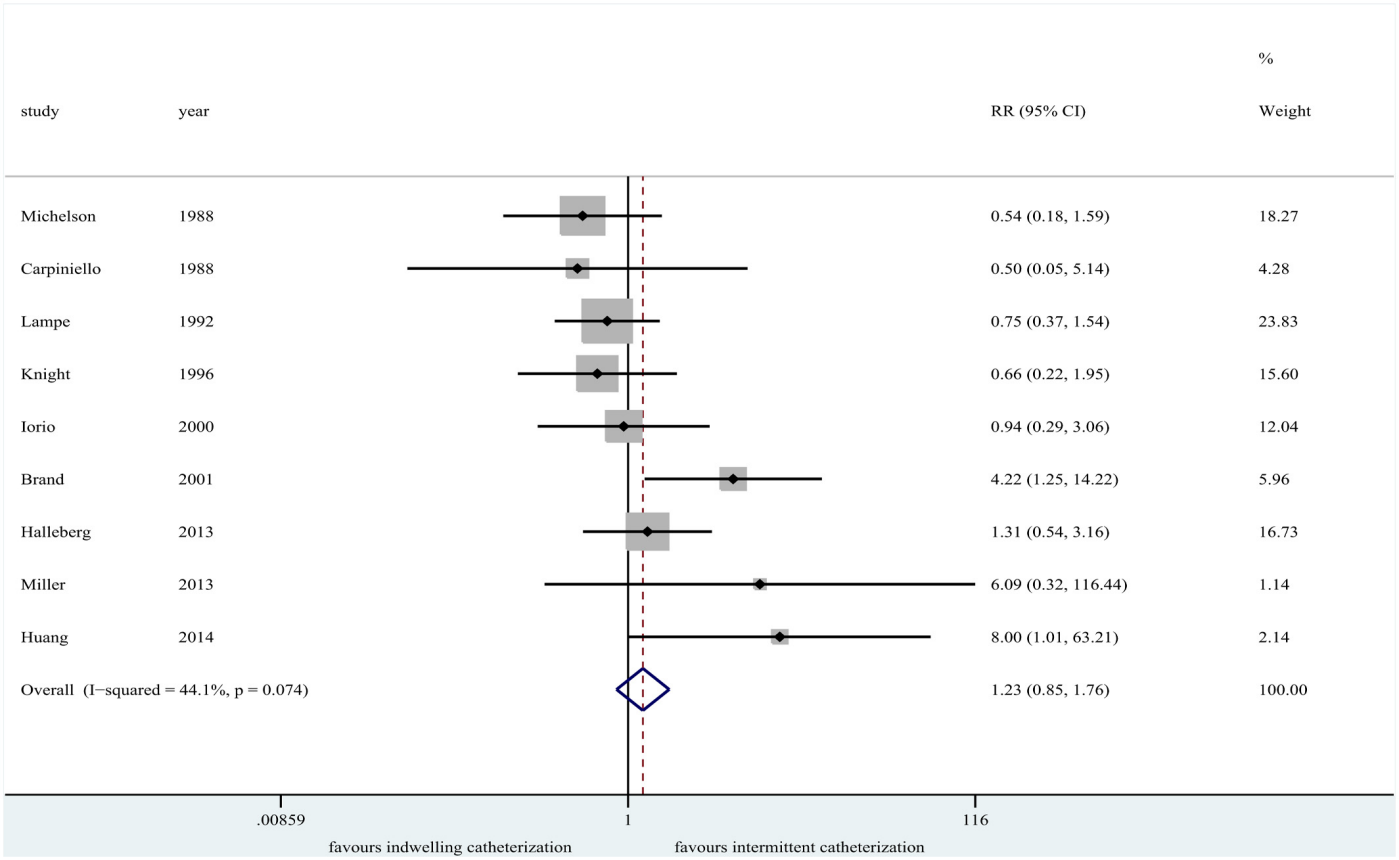
Forest plot for urinary tract infection.

**Fig 3:**
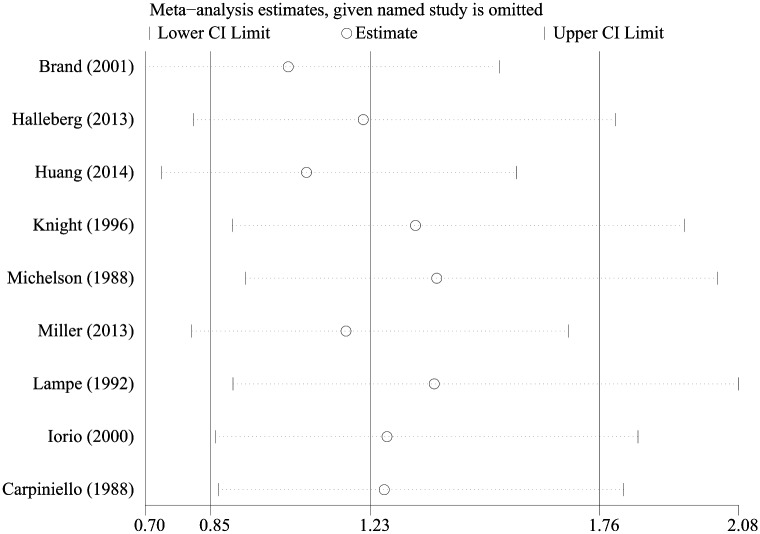
Sensitivity analyses of urinary tract infection.

UTI events were recorded in all the included studies. No significant difference was detected between indwelling catheterization group and the intermittent catheterization group (*n* = 1771; RR = 1.23, 95% CI [0.85, 1.76], *P* = 0.268; *I* ^*2*^ = 44.1%, *P* = 0.074; Fig 2). Moderate heterogeneity was demonstrated in the pooled result (*I* ^*2*^ = 44.1%). To identify the origin of the heterogeneity among the studies, a meta-regression analysis was conducted, based on study methodological quality (high and moderate quality), study region, publication year, and the duration of indwelling catheterization. Nonetheless, those factors were apparently not the origin of the heterogeneity among studies (*P*>0.05; S2 Table). Then, a sensitivity analysis was also performed (Fig 3). When Brand et al.’s data [12] were excluded, the heterogeneity decreased significantly from 44.1% to 18%. However, there was no difference in risk of UTI between the two groups (*n* = 1672; RR = 1.03, 95% CI [0.70, 1.51], *P* = 0.77; *I* ^*2*^ = 18%, *P* = 0.29).

**Fig 4:**
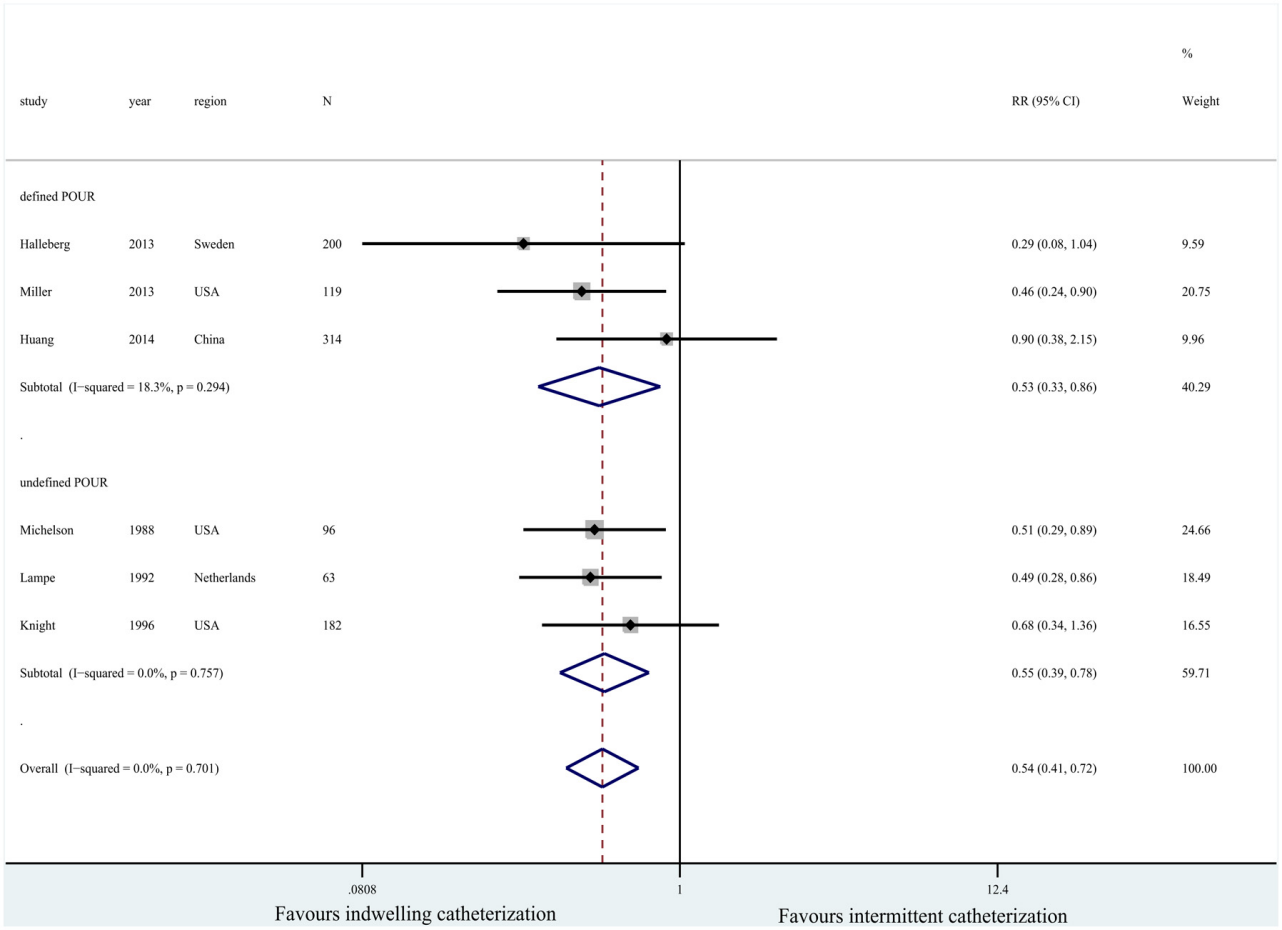
Forest plot for postoperative urinary retention.

Six studies reported POUR events. Of them, three [8, 9, 17] did not provide a specific definition of POUR, and the others defined it as the inability to void when a bladder volume was >400 mL [10, 14, 15]. Accordingly, in a subgroup analysis, these were divided into two groups: the defined POUR group and the undefined POUR group. A forest plot showed that indwelling catheterization reduced the risk of POUR compared with intermittent catheterization following total joint arthroplasty (*n* = 974; RR = 0.54, 95% CI [0.41, 0.72], *P*<0.00001; *I* ^*2*^ = 0%, *P* = 0.701; Fig 4).

Depending on the included studies spanned a longer period, patients would have been treated over a 20-year period. Today, patients are mobilized earlier and treated with more contemporary pain management strategies than those of the 1990s, which may influence the occurrence of POUR. Thus, we next performed a stratified analysis based on the publication year to compare studies from before and after 2000. The pooled results also showed that fewer patients experienced POUR if they were treated with indwelling catheterization (*n* = 974; RR = 0.54, 95% CI [0.41, 0.72], *P*<0.00001; *I* ^*2*^ = 0%, *P* = 0.701; Fig 4).

**Fig 5:**
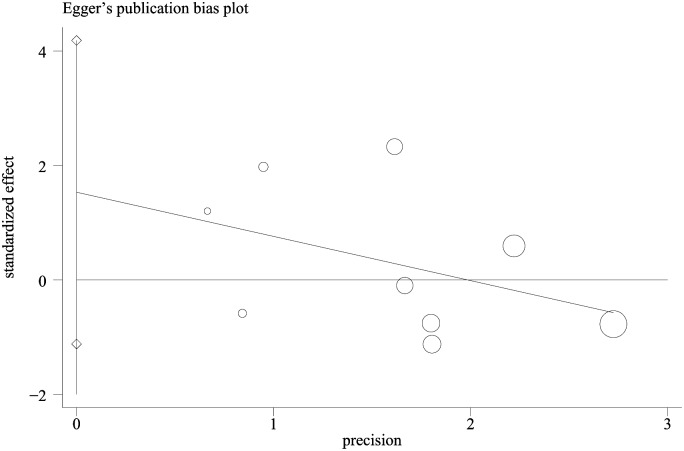
Egger’s publication bias plot.

The results of the Begg’s test (*P* = 0.175, continuity corrected) and the Egger’s test (*P* = 0.278; Fig 5) showed that there was no publication bias in this study.

## Discussion

To our knowledge, this study is the first meta-analysis that quantitatively analyzes and summarizes the risks of UTI and POUR following total joint arthroplasty after indwelling or intermittent catheterization. However, it has the following limitations. First, to date, there is no precise definition of POUR, even among urologists [10]. Thus, in our study, the definition of POUR might not be consistent, which may have resulted in bias and weakened our analysis. However, we performed a subgroup analysis of this. Second, a small number of RCTs was included in this study. Thus, we were unable to stratify according to surgical site or gender, although we excluded publication bias and proved that the pooled outcomes were stable. Third, four RCTs [8, 9, 17, 18] did not report the conditions of anesthesia. We were unable to identify whether the two groups were comparable with respect to the use of opiates and antibiotics, as most authors did not report sufficient data. These factors might have affected the results, which might result in bias. Furthermore, the publication year of RCTs included in our study spanned a long period (1988–2014), which could also have lead to bias. However, we conducted a meta-regression analysis to assess confounding factors, and no significant determinant was found (*P*>0.05).

The most important finding of the meta-analysis was that indwelling catheterization with removal 24–48 h postoperatively decreased the rate of POUR, and did not increase the risk of UTI.

Regarding POUR, we found that indwelling urinary catheterization reduced the risk of POUR compared with intermittent catheterization (RR = 0.5). Similar results were reported previously [2, 8, 9, 11, 26]. In our study, the average age was 64.5 years, which might be related to urethral mechanical obstruction caused by prostatic hypertrophy, as well as age-related progressive neuronal degeneration resulting in bladder dysfunction [27]. Anesthesia and other factors might result in a decrease in or absence of bladder sensation, causing delayed interventions in urinary dysfunction. The functional recovery of the bladder is based on whether reversible or irreversible injury has occurred during the overdistension period [4]. Nevertheless, the indwelling catheterization inevitably decompresses the bladder, and it could prevent bladder injury effectively [4]. Moreover, several studies have also confirmed that intermittent catheterization resulted in a higher risk of repeated bladder distension above 700 mL [8, 17].

Theoretically, in patients, prolonged use of urinary catheters could be associated with UTI [17, 28]. However, rates of UTI were similar between an indwelling catheter, for 24–48 h, and intermittent catheterization following lower total joint arthroplasty in this study. Intermittent urinary catheterization has been associated with a risk of bladder overdistension, which may cause permanent impairment of detrusor function and thereby increase the volume of post-voiding residue [2]. Overdistension of the bladder and large static post-void residual volumes might increase susceptibility to bladder infection [8]. Moreover, multiple catheter insertions might damage the mucosal barrier, possibly having an additive effect on the cumulative risk of UTI [17].

Several studies have suggested that catheterizations could be avoided in some patients through not using indwelling catheterization routinely [6, 15, 29]. Avoidance of continuous urinary catheterization is also associated with earlier activity and functional recovery [29]. Consequently, combined with our results, the risk of POUR should be considered in deciding whether to perform indwelling catheterization before surgery. The International Prostate Symptom Score has been proposed to predict the risk of POUR, because it quantifies lower urinary tract symptoms [7]. In patients with multiple risk factors for POUR, including males [30, 31], age >70 years [7, 32], benign prostatic hypertrophy [14], positive urologic anamnesis [31], opiate consumption [10, 30], and the operative time >100 min [30], indwelling urinary catheterization with removal 24–48 h postoperatively may be the preferred choice. For patients without a high risk of POUR, either indwelling catheterization or intermittent catheterization could be appropriate. However, considering that some patients could avoid catheterizations following the surgery, intermittent catheterization may be a good option.

## Conclusions

Based on available evidence, indwelling urinary catheterization with removal 24–48 h postoperatively was superior to intermittent catheterization in preventing POUR. Furthermore, it did not increase the risk of UTI. For patients with multiple risk factors for POUR undergoing the lower total joint arthroplasty, indwelling urinary catheterization with removal 24–48 h postoperatively is a valid and reasonable option. Given relevant possible biases in our study, adequately powered and better-designed RCTs are required to elucidate amore objective conclusion.

## Supporting Information

S1 PRISMA Checklist PRISMA 2009 Checklist. (DOC)

S1 Table Search strategy for PubMed. (DOCX)

S2 Table Meta-regression for variables that influence the association for the pooled results of UTI. (DOCX)

S1 Text Search strategy for Embase. (DOCX)
