# Supplementary material for: Indwelling versus Intermittent Urinary Catheterization following Total Joint Arthroplasty: A Systematic Review and Meta-Analysis
Source: PLoS One. 2015 Jul 6;10(7):e0130636. doi: 10.1371/journal.pone.0130636 (PMC4492963; doi:10.1371/journal.pone.0130636)
Supplement: S1 Table — (DOCX) [file pone.0130636.s002.docx]

| **Search strategies in Pubmed: #3** |
| --- |
| #3  Search (((((((((((((total knee arthroplasty[Title/Abstract]) OR total knee replacement[Title/Abstract]) OR total hip replacement[Title/Abstract]) OR total hip arthroplasty[Title/Abstract]) OR TKA[Title/Abstract]) OR TKR[Title/Abstract]) OR THR[Title/Abstract]) OR THA[Title/Abstract]) OR total joint replacement[Title/Abstract]) OR total joint arthroplasty[Title/Abstract]) OR TJA[Title/Abstract]) OR TJR[Title/Abstract])) AND (((catheter[Title/Abstract]) OR catheterization[Title/Abstract]) OR catheterize[Title/Abstract])  #2  Search (((((((((((total knee arthroplasty[Title/Abstract]) OR total knee replacement[Title/Abstract]) OR total hip replacement[Title/Abstract]) OR total hip arthroplasty[Title/Abstract]) OR TKA[Title/Abstract]) OR TKR[Title/Abstract]) OR THR[Title/Abstract]) OR THA[Title/Abstract]) OR total joint replacement[Title/Abstract]) OR total joint arthroplasty[Title/Abstract]) OR TJA[Title/Abstract]) OR TJR[Title/Abstract]  #1  Search ((catheter[Title/Abstract]) OR catheterization[Title/Abstract]) OR catheterize[Title/Abstract] |
|  |
|  |
