# Supplementary material for: Indwelling versus Intermittent Urinary Catheterization following Total Joint Arthroplasty: A Systematic Review and Meta-Analysis
Source: PLoS One. 2015 Jul 6;10(7):e0130636. doi: 10.1371/journal.pone.0130636 (PMC4492963; doi:10.1371/journal.pone.0130636)
Supplement: S2 Table — (DOCX) [file pone.0130636.s003.docx]

**Meta-regression for variables that influence the association for pooled result of UTI.**

| Variables | Study variance (tau^2^) | *P* value | Residual variation due to heterogeneity (*I^2^*) |
| --- | --- | --- | --- |
| Publication year | 0.089 | 0.058 | 11.46% |
| Region | 0.155 | 0.095 | 27.62% |
| Duration of indwelling catheterization | 0.3033 | 0.574 | 45.80% |
| Study quality | 0.3983 | 0.604 | 48.02% |
