# Supplementary material for: Indwelling versus Intermittent Urinary Catheterization following Total Joint Arthroplasty: A Systematic Review and Meta-Analysis
Source: PLoS One. 2015 Jul 6;10(7):e0130636. doi: 10.1371/journal.pone.0130636 (PMC4492963; doi:10.1371/journal.pone.0130636)
Supplement: S1 Text — (DOCX) [file pone.0130636.s004.docx]

**Embase search strategy**

**#3** **#1** combined **#2**

**#2** 'catheterization' OR 'catheter' OR 'catheterize'

**#1** 'total knee arthroplasty'/exp OR 'total knee arthroplasty' OR 'total knee replacement'/exp OR 'total knee replacement' OR 'total hip arthroplasty'/exp OR 'total hip arthroplasty' OR 'total hip replacement'/exp OR 'total hip replacement' OR 'total joint arthroplasty' OR 'total joint replacement'/exp OR 'total joint replacement'
